# A Self-Calibrated Single Wavelength Biosensor for Measuring Oxygen Saturation

**DOI:** 10.3390/bios14030132

**Published:** 2024-03-04

**Authors:** Michal Katan, Ori Pearl, Alon Tzroya, Hamootal Duadi, Dror Fixler

**Affiliations:** 1 Faculty of Engineering, Bar Ilan University, Ramat Gan 5290002, Israel; michal.katan@live.biu.ac.il (M.K.); alon.tzroya@biu.ac.il (A.T.); hamootal.duadi@biu.ac.il (H.D.); 2 The Institute of Nanotechnology and Advanced Materials, Bar Ilan University, Ramat Gan 5290002, Israel

**Keywords:** biosensor, oxygen saturation, scattering, light-tissue interaction, tissue diagnostics optics

## Abstract

Traditional methods for measuring blood oxygen use multiple wavelengths, which produce an intrinsic error due to ratiometric measurements. These methods assume that the absorption changes with the wavelength, but in fact the scattering changes as well and cannot be neglected. We found that if one measures in a specific angle around a cylindrical tissue, called the iso-pathlength (IPL) point, the reemitted light intensity is unaffected by the tissue’s scattering. Therefore, the absorption can be isolated from the scattering, which allows the extraction of the subject’s oxygen saturation. In this work, we designed an optical biosensor for reading the light intensity reemitted from the tissue, using a single light source and multiple photodetectors (PDs), with one of them in the IPL point’s location. Using this bio-device, we developed a methodology to extract the arterial oxygen saturation using a single wavelength light source. We proved this method is not dependent on the light source and is applicable to different measurement locations on the body, with an error of 0.5%. Moreover, we tested thirty-eight males and females with the biosensor under normal conditions. Finally, we show the results of measuring subjects in a hypoxic chamber that simulates extreme conditions with low oxygen.

## 1. Introduction

BioConvergence is a rapidly emerging field that involves the integration of various disciplines, including biology, engineering, physics, and computer science, to develop innovative solutions for healthcare problems [1]. Bio-devices, including medical devices, are designed to interact with biological systems for the purpose of diagnosing, monitoring, or treating health conditions [2]. Bio-chips are a subset of bio-device technologies, and are usually equipped with sensors and actuators that detect biological conditions and respond to changes in the biochemical environment. The integration of BioConvergence technologies with bio-devices and bio-chips has the potential to revolutionize the field of medicine by providing more effective monitoring tools or innovative treatments [3]. 

Optical bio-devices for diagnosis of physiological states, refer to a class of bio-devices that combine optics and biological interactions, and have several advantages over traditional biochemical methods, including high sensitivity, speed, and non-invasiveness [4]. Unfortunately, human tissue poses a challenge due to its turbidity, therefore in the field of optics the focus is on sensing changes in absorption. The reemitted light from the tissue is dependent inseparably on both scattering and absorption. While scattering is mostly influenced by the tissue structure, the absorption is mainly affected by the biochemical composition of the tissue [5]. In order to extract physiological parameters from absorption, such as blood saturation, one must assume, manipulate, or neglect values of the scattering. This assumption is required due to the strong dependency of the light intensity on scattering and absorption.

Oxygen saturation is defined as the proportion of oxygenated hemoglobin in the total hemoglobin concentration. The oxygen saturation level indicates the ability of the body to provide a sufficient amount of oxygen to all organs and reflects the proper or improper functioning of the lungs. Normal values of arterial oxygen saturation (SpO_2_) are considered to be between 95–100% for adults, while values below 90% may indicate hypoxia or hypoxemia [6]. 

Optic methods for extracting oxygen saturation are based on detecting the photoplethysmogram (PPG) waveform, an optical signal that reflects the change in blood volume through the tissue [7]. In fact, the PPG signal represents the reemitted light intensity from the tissue and is composed of constant and varying components. The constant component of the PPG waveform depends on the tissue structure, such as bone, fat, melanin, as well as non-pulsatile arterial and venous blood. On the other hand, the varying component is composed of the arterial blood fluctuations. The ratio between the pulsatile and non-pulsatile components leads to an absorption component that is proportional to arterial blood and optical pathlength, from which the oxygen saturation can be derived [8]. 

Nowadays, the common method for characterizing oxygen saturation is pulse oximetry. This method uses at least two different wavelengths, red and infrared, for measuring the ratio between oxygenated hemoglobin and deoxygenated hemoglobin [9]. At each wavelength there is a different optical pathlength (OPL), in accordance with different unknown reduced scattering coefficients. The classic method neglects the differences in OPL or tries to assess them [10] to compensate for the interdependence of the scattering and absorption [11], which produces an intrinsic system error. 

Existing methods to extract oxygen saturation include measurements using multiple wavelengths [12,13,14], averaging multiple sensors [15], measuring behind the ear [16], and remote sensing measurements [17,18,19]. Both classical methods and the methods mentioned previously have errors ranging between 2–3% and are still insensitive outside of the normal saturation range. 

The work we present in this paper is based on a physical phenomenon that was revealed in our lab, called the iso-pathlength (IPL) point [20,21,22,23]. The IPL point is an angle around a cylindrical tissue, such as finger or wrist, that is invariant to scattering. This was discovered by a new method of measuring the full scattering profile (FSP), which is the angular distribution of light intensity of cylindrical tissues [21]. For this reason, at the IPL point two different wavelengths experience the same optical pathlength in biological tissue condition, which solves the inherent error of assessing oxygen saturation. Another option, which we implement in this work, is using a single wavelength source and a few angles, while the IPL point serves for self-calibration. This phenomenon was verified in finger-sized tissue-mimicking phantoms as well as human fingers [24]. It was also found that there is a linear relationship between the diameter of the cylindrical tissue and the angle at which the IPL point appears [25]. By using these principles, we designed an optic biosensor for reading the light intensity reemitted from the tissue, which uses a single light source and multiple photodetectors (PDs), with one of them in the IPL point’s location. For the first time in the field of oxygen saturation, the concept of the IPL point enables measuring subjects’ SpO_2_ with respect to another position on the tissue rather than another wavelength and external calibration. The IPL point’s angle, as estimated in lab experiments, is relatively close to the light source, and can be easily translated into a distance. We will present the results of nearly 40 subjects, both males and females, that had their oxygen saturation measured.

## 2. Materials and Methods

### 2.1. Theory

The FSP of the transmitted light, *I_t_*, is a function of absorption, scattering, wavelength, OPL and the geometry of the tissue [21]. More specifically, for a cylindrical tissue with an absorption coefficient *μ_a_* and a reduced scattering coefficient *μ’_s_*, the light intensity at an angle *θ* is given by the Beer–Lambert law:

(1) $$I_{t}\left(\theta ,\;\mu_{a},\mu \prime_{s}\right)=I_{0}\left(\theta ,\mu \prime_{s}\right)\exp\left[-\mu_{a}\cdot l\left(\theta ,\;\mu_{a},\mu \prime_{s}\right)\right]$$

where *I*_0_ is the light intensity without absorption and *l* is the OPL. The major problem is that the OPL as well as the absorption and reduced scattering coefficients are unknown and depend on the optical properties of the tissue.

**Figure 1:**
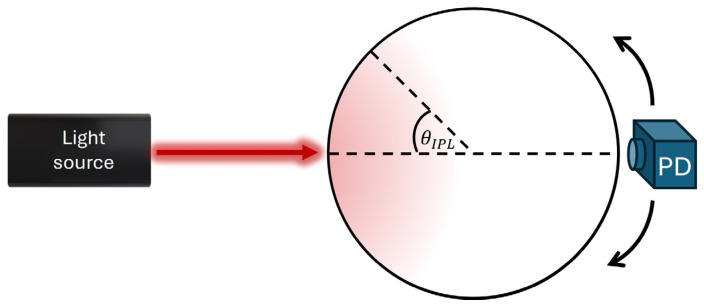
Illustration of the optical system for measuring the iso-pathlength (IPL) point. A photodetector (PD) collects the reemitted light intensity from all angles around the sample, called the full scattering profile (FSP). The IPL point, denoted as *θ_IPL_* in the figure, is a specific position on the circumference of the sample.

The IPL point (as illustrated in Figure 1) is a geometrical point on a cylindrical tissue’s surface, which depends only on the diameter [26]. At this point, the light intensity and the OPL are constant regardless of variations in tissue scattering. We define R to be the ratio between the intensities at the IPL point with and without absorption. The differential pathlength factor (DPF) is the ratio between the OPL and the distance between the light source and the PD, d. With DPF_0_ defined as the DPF in the case of no absorption, the absorption coefficient can be extracted, as defined in [26]:

(2) $$\mu_{a}=-\frac{\ln\left(R\right)}{d\cdot DPF_{0}\cdot \sqrt{R}}$$

The absorption coefficient of human tissue has been widely investigated. As described in Steven Jacques’ review [27], the absorption coefficient of a generic tissue is a superposition of all its parts: blood, fat, melanin, bone, and so on. The equation is denoted as the sum of the absorption coefficient of each component of the tissue, *μ_a,i_* [m^−1^], multiplied by the volume fraction of the component, *f_v,i_*:

(3) $$\mu_{a}=\underset{i}{\sum }f_{v,i}\cdot \mu_{a,i}$$

For the range of wavelengths, usually red and infrared, used by methods typically intended for extracting oxygen in blood, the most significant absorptive components are oxygenated and deoxygenated hemoglobin (*μ_ox_* and *μ_dox_*), as well as melanin, which will be neglected. For this reason, Equation (3) can be adjusted to:

(4) $$\mu_{a}^{blood}=\left(C_{p}+\Delta C_{p}\right)\left[\mu_{ox}SpO_{2}+\mu_{dox}\left(1-SpO_{2}\right)\right]+C_{v}\left[\mu_{ox}SvO_{2}+\mu_{dox}\left(1-SvO_{2}\right)\right]$$

**Figure 2:**
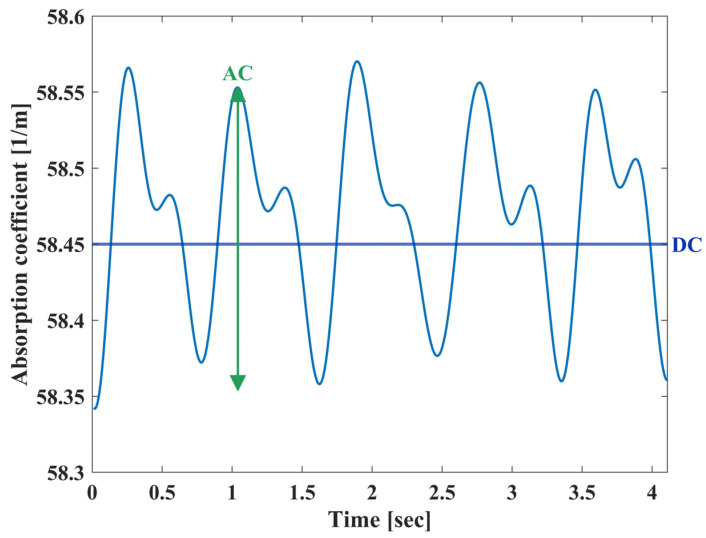
The photoplethysmogram (PPG) signal as extracted from the optic biosensor. The PPG waveform is a periodic signal with both higher and lower peaks, representing the systolic and diastolic phases, through the cardiac cycle. The signal consists of an AC component, influenced by the blood fluctuations, and a DC component, derived from the non-pulsatile elements in blood and tissue.

SpO_2_ and SvO_2_ represent the oxygen saturation in arterial and venous blood, respectively. ΔC_p_ and Cp are the pulsatile or non-pulsatile portions of the artery blood, while C_v_ represents the non-pulsatile portion of venous blood. We define *μ_a_*^75%^ to be the absorption coefficient when the average of SpO_2_ and SvO_2_ is set to be 75%, as described in [27]. *μ_a_*^75%^ is composed from the constant elements of both arterial and venous blood, and therefore can be described as a DC component (Figure 2). The pulsatile component, denoted as AC, depends only on the SpO_2_.

In order to extract the arterial blood saturation, we follow the common method of examining the ratio between pulsatile and non-pulsatile components of the blood [7,8,12,28,29]. In contrast to the classic method, which uses light intensity, we work with the absorption coefficients presented in Equation (2). The AC/DC ratio can be described by the pulsatile component in Equation (4), and the DC is represented by *μ_a_*^75%^:

(5) $$\frac{\mu_{a}^{AC}}{\mu_{a}^{DC}}=\frac{\Delta C_{p}\left(\mu_{ox}SpO_{2}+\mu_{dox}\left(1-SpO_{2}\right)\right)}{\mu_{a}^{75\%}}-C$$

At the end of diastole, when the blood fluctuations are minimal and the AC component is approximately zero, there is still a DC component. Therefore, in order to force the oxygen saturation trendlines to cross the origin, the equation also includes an empirical factorial correction, C. To further simplify the equation, let us mark F = ΔC_p_/*μ_a_^75%^*. Equation (6) illustrates the final extraction of SpO_2_, which we implemented in the biosensor measurements.

(6) SpO2=μaACFμaDC+C−μdoxμox−μdox

### 2.2. Biosensor

**Figure 3:**
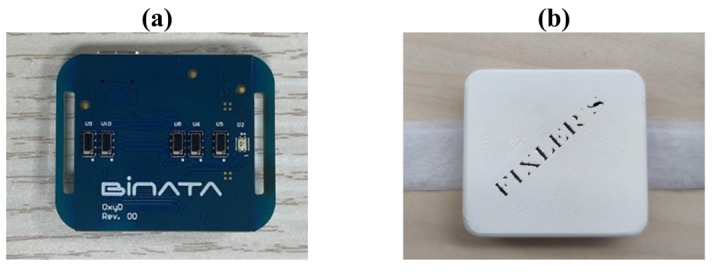
(**a**) The interior of the biochip, equipped with five photodetectors (PDs) and a single wavelength LED. (**b**) The chip inside its protective case.

In this study we designed an optic biosensor for measuring oxygen saturation by utilizing the IPL point. The biosensor was manufactured according to our design by BINATA (Yokneam, Israel). The chip consists of a red LED with a wavelength of 655 [nm] to fit the absorption range of hemoglobin [27] and five PDs located at different distances from the light source (Figure 3a). From these five PDs, two are used to calculate the SpO_2_ from the ratio between them, with one of the PDs being used specifically for self-calibration. The chip operates at a frequency of 100 [Hz] to fit the necessary sampling rate for a PPG signal [30]. The PPG signal is known to be affected by ambient light; therefore, the biosensor is equipped with a component that modulates the LED and PDs in the same frequency and performs synchronized detection (ADPD4101, Analog Devices, Norwood, MA, USA). The PDs of the sensor collect the reemitted light intensity from the tissue simultaneously, which helps tackle movement artifacts. The light measured by each PD is optimized to find the IPL point’s location per subject, thus operating as a self-calibrating sensor. The chip is equipped with a plastic cover printed by a 3D printer that protects the PDs from directly touching the skin and allows comfortable use (Figure 3b).

### 2.3. Phantoms

We tested the biosensor on tissue-mimicking phantoms we produced in the lab, in order to inspect the sensor. The phantoms are made of a PDMS and TiO_2_ mixture that underwent a degassing process [31]. We measured four phantoms with different reduced scattering coefficients, in the range of 16–26 [1/cm], since this range represents human skin [27].

### 2.4. Human Research

Following the phantoms’ measurements, we advanced to measuring human wrists using the biosensor. We measured 38 adults, all between the ages of 17–77, both males and females as described in Table 1 (all subjects signed a participant consent form). Each subject was measured twice using the sensor, at the internal and external side of the wrist, with each test lasting about 60 s. The average of both measurements is shown in the last column of Table 1. All subjects were requested to sit comfortably without moving or talking throughout the entire measurement. Furthermore, we tested their oxySgen saturation with a pulse oximeter (CMS50M, Contec Medical Systems, Qinhuangdao, China) as a reference device in order to compare the findings. Next, we tested several subjects under abnormal conditions in the hypoxic chamber in the Wingate institute (Netanya, Israel) [32].

### 2.5. Processing Analysis

**Figure 4:**
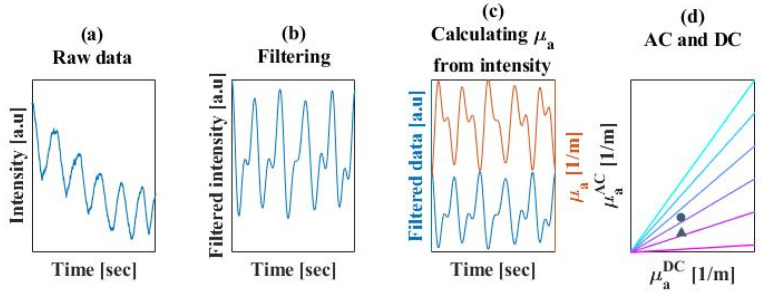
Processing stage. (**a**) The raw data extracted from the biosensor, meaning the reemitted light intensity from the tissue collected by one of the PDs over time. (**b**) The data post filtered between 1–3 [Hz]. The received signal is a reversed PPG waveform, since the light intensity is proportionally inverse to the blood pumping through the arteries. (**c**) The filtered intensity converted into absorption coefficients, resulting in a corrected PPG waveform. (**d**) The extracted AC and DC absorption values from the PPG signal with the colored lines representing different oxygen saturation, between 95–100%. Measured values of the oxygen saturation are shown on the graph, with results from the inner side of the wrist marked with a circle and the external side of the wrist marked with a triangle.

After extracting the raw data from the device, we began the processing stage (Figure 4). First, we transferred the signal to the frequency domain using the Fourier transform (Figure 3a). Then, we filtered the relevant frequency: between 1 [Hz] and 3 [Hz], as commonly performed on PPG data [30,33] (Figure 4b). The filtering also helps clean irrelevant noise and motion artifacts. Then we could transfer back to the time domain and convert the filtered data into absorption coefficients, using Equation (2) (Figure 4c). I_0_ is the light intensity without absorption, measured from the phantoms. It is the light intensity measured from the tissue by the biosensor, d is the distance between the light source and the detector at the IPL point, and DPF_0_ is set to be 12, as described in [26]. The next step was selecting the PPG shapes, and then oxygen saturation could be calculated according to the ratio between AC and DC as described in Equation (6) (Figure 4d). The traditional methods continued from the filtering step right to the AC/DC observation, without extracting absorption coefficients, and used two wavelengths instead of one in order to find a signal that is proportional to *μ*_a_.

## 3. Results

### 3.1. Biosensor’s Measurement of Phantoms

**Figure 5:**
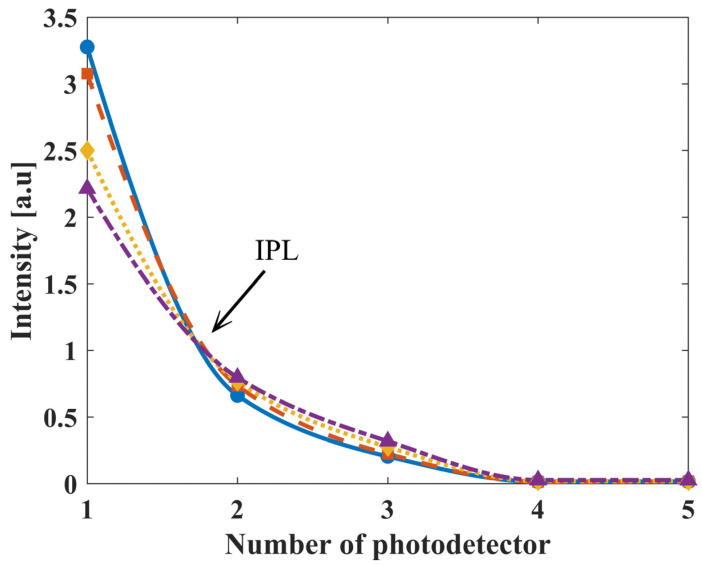
The light intensity measured by the biosensor of four PDMS phantoms with different reduced scattering coefficients. The phantoms have a *μ*’*_s_* value of 17, 19, 24 and 28 [1/cm] represented by a blue solid, orange dashed, yellow dotted, and purple dash-dotted line, respectively. There is an intercept slightly before the second PD, representing the location of the IPL point.

First, we measured four phantoms with the biosensor, each with different reduced scattering coefficients (Figure 5). The biosensor’s PDs in this experiment represent the different locations around the phantom. This indicates and further proves the existence of the IPL point, where the second PD is located. In previous studies we have discussed the influence of system errors, as well as the influence of detector size, distance from the tissue, etc. [25,26]. Nevertheless, given all these errors, we claim that this method is still viable, with this work being the first proof of concept.

### 3.2. Extracted Saturation Values of Healthy Population

**Figure 6:**
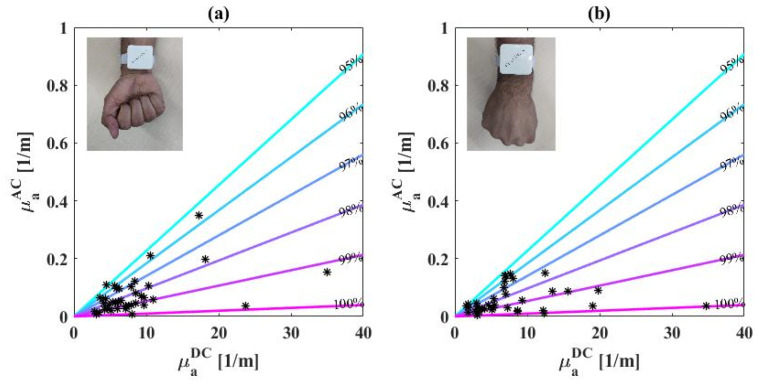
AC vs. DC absorption values of all subjects’ inner wrists (**a**) and external (**b**) sides. The colored lines represent the normal values of oxygen saturation (95–100%) and the asterisks represent the measured values per subject. The insets show the location of the sensor on the wrist.

Figure 6 shows the results of 38 subjects that were tested with the biosensor on their wrists. As described in the methods section, all subjects were tested on the inner and outer sides of their wrists, as shown in the inset of the graph. The plot compares the AC to DC for all measurements, while the colored lines represent oxygen saturation in the range of 95–100%. The oxygen saturation of all subjects is calculated to be in the range of 95–100% for the inner side of the wrist, and 94.8–100% for the external side of the wrist. Most measurements are between 97–99% which is as expected since this is the range of saturation for healthy people. The DC measured in the inner wrist appears to be concentrated in the smaller values of the axis, compared to the external wrist where the DC seems to be more spread-out over the axis. This result matches with the fact that naturally there is a higher amount of melanin on the external side of the wrist, which has strong absorption.

**Figure 7:**
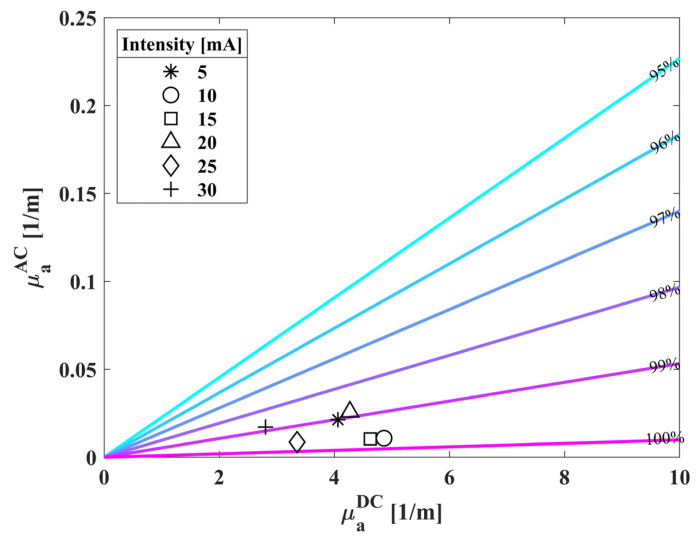
AC vs. DC absorption values of a single individual. We measured the subject’s wrist several times while changing the biosensor’s LED intensity to evaluate the standard deviation between saturation measurements. The colored lines represent the normal values of oxygen saturation (95–100%).

We also wanted to determine whether the biosensor’s light source intensity has an influence on the measurements. Figure 7 shows the calculated oxygen saturation, conducted on a single individual, with different intensities applied to the biosensor’s LED, 5–30 [mA] every 5 [mA]. The colored lines represent the oxygen saturation of 95–100% as previously described in Figure 6. The average value of this experiment is 99.3% with a standard deviation of 0.4%, meaning the biosensor has an error of less than half a percentage.

**Figure 8:**
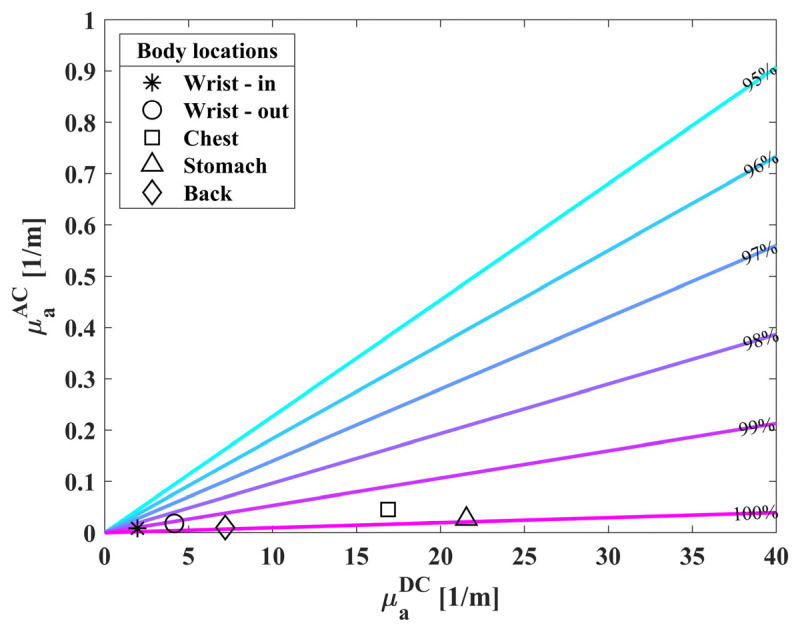
AC vs. DC absorption values of a single individual. We measured the subject at different locations on the body: the internal and external sides of the wrist, chest, stomach and back. The colored lines represent the normal values of oxygen saturation (95–100%).

Next, we placed the biosensor at different locations on a single subject: the internal and external sides of the wrist, the chest, the lower back, and the stomach. These locations were chosen under the low penetration depth of red light and are possible locations to assess the skin microcirculation [34,35]. Chest, stomach and back may not be cylindrical, but can be considered as semi-infinite samples, thus the IPL point concept is valid even in these cases [36]. For each location we measured the oxygen saturation for 60 s, in order to test the influence of location on the biosensor, as described in Figure 8. It is clear that all locations produce a similar value, and the calculated mean value is 99.8% with a standard deviation of 0.4%.

**Figure 9:**
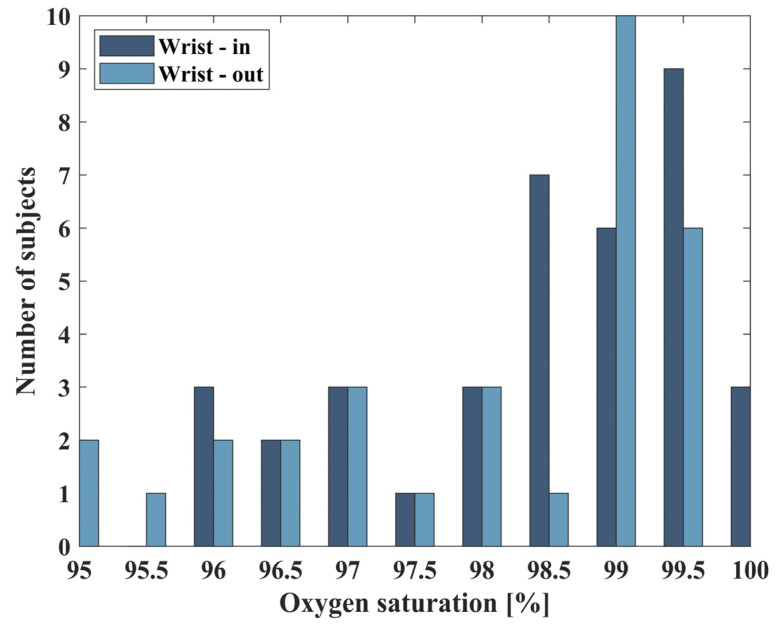
The number of subjects per measured oxygen saturation from the internal and external sides of their wrists, in steps of 0.5%.

We also compared the extracted oxygen saturation of all 38 subjects, for both the internal and external sides of the wrist (Figure 9). As expected, almost all subjects measured had saturation within the range between 95% to 100%, while most measurements lie on the right side of the graph, between 98.8% to 100%. The calculated mean value between individuals is 98.4% for both sides with a standard deviation of 1% for the inner side and 2% for the external side of the wrist.

### 3.3. Extracted Saturation Values in Hypoxic Conditions

**Figure 10:**
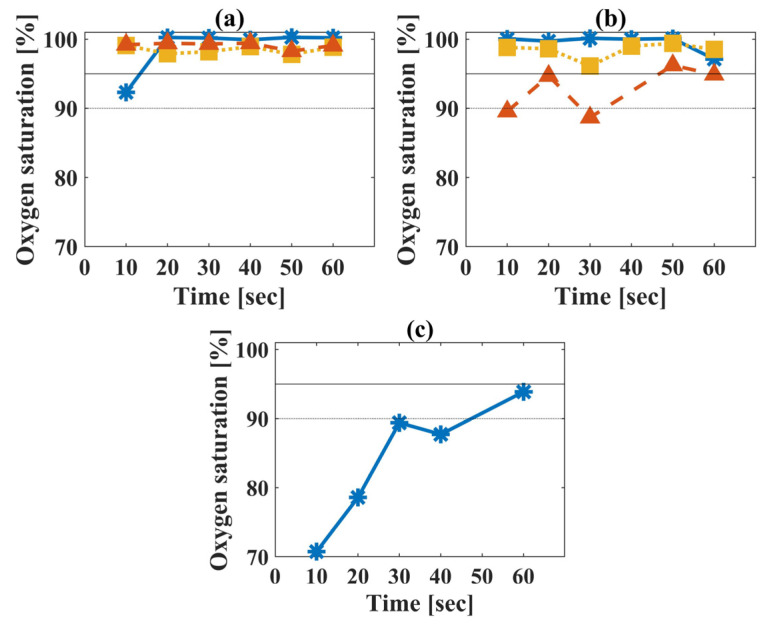
Results of the subjects’ oxygen saturation over time, where each test took 60 s. (**a**) The male subjects were measured right after they entered the hypoxic chamber (**b**) and after exercising. The three of them are represented by a blue solid, orange dashed and dash-dotted yellow line, in both graphs. (**c**) The female subject was tested right after exiting the room. The female’s results show a significantly low oxygen saturation, corresponding with the subject’s report of feeling unwell.

As a part of this study, we conducted an experiment where subjects were placed in a hypoxic chamber that reduced the oxygen in the air [32]. The oxygen level inside the chamber was approximately 14.5%, which is equivalent to a height of 3000 m above sea level [37], in contrast to a normal oxygen percentage of 21% at sea level. We tested four subjects, three males and one female, with each measurement lasting 60 s per subject. According to the operators of the hypoxic chamber, the typical reaction when entering the chamber is that the subjects’ oxygen levels decline, then immediately stabilize back to normal. To overcome the fast recovery of the body and to decrease the oxygen saturation, each one of the male subjects was asked to exercise inside the hypoxic chamber. Figure 10a,b demonstrate the results of the male subjects, before and after exercising, respectively. Our results indicate that the oxygen saturation of the male subjects remained quite high, save for one. 

The female subject reported feeling unwell whilst inside the chamber. Therefore, she was not asked to exercise, and was measured right after exiting the hypoxic chamber. The measurement produced a significantly lower saturation level (Figure 10c), which corresponded with the general ill feeling of the subject and her appearance.

## 4. Discussion

The optical measurements of a human tissue depend on both scattering and absorption, and are inseparable. In the field of bio-optical sensing of physiological parameters, the focus is on sensing absorption due to the blood’s property of being highly absorptive. Nowadays, this field lacks any methods for isolating absorption from scattering, therefore for measuring blood oxygen, most methods use multiple wavelength measurements which require additional calibration, resulting in inherent errors. By using the IPL point, we developed a method for quantitating the absorption from the reemitted light intensity from the tissue. We designed a sensor equipped with a single wavelength light source and several PDs, based on the IPL point’s concept. For this reason, the biosensor calibrates itself per subject and does not require further calibration. Therefore, the sensor lacks the traditional inherent errors related to common SpO_2_ sensors.

The standard deviation of an oxygen saturation measurement through a common pulse oximeter is claimed to be ±2%, while the standard deviation from measuring with our biosensor was demonstrated to be less than ±0.5%, when comparing the different measurements performed on a single test subject. The biosensor’s results show its independence from the light source, since different LED’s intensities produced similar values of oxygen saturation, with an error of 0.4%. The same error was also obtained when testing on different locations on the body of a single subject.

**Figure 11:**
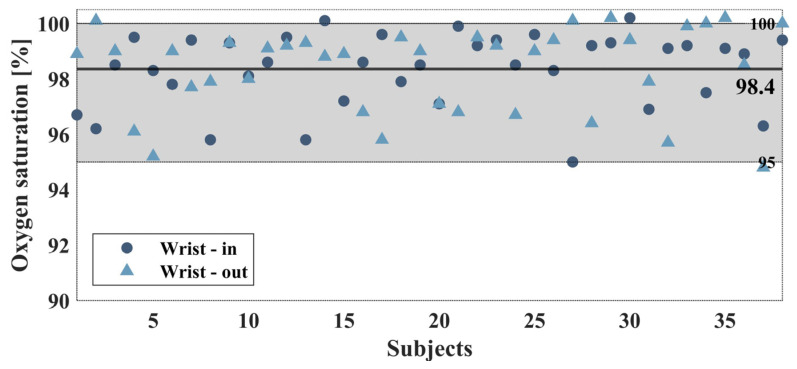
Summary of the extracted oxygen saturation of all 38 subjects, where the grey region denotes the normal values of saturation, between 95% to 100%, with the expected result that nearly all subjects measured had a saturation that lay within this range.

The experiment included the participation of 38 subjects, with measurements taken from both their inner and outer wrist. Figure 11 summarizes the oxygen saturated measured from all subjects, with the grey region denoting the normal values of saturation. For half of all participants (16 of all 38 subjects), the oxygen saturation in both sides of the wrist was calculated with less than 1% difference. Among the remaining 22 subjects that were tested with more than 1% difference, 13 of them had a low DC value for at least one of the wrist sides. Low DC represents having an absorption of less than 5 [m^−1^] (Figure 6). These low values of DC indicate that the LED’s intensity is overly high and thus reaches the saturation of the sensor. This can be amended by altering the intensity during the measurement and allows for improved results.

The new self-calibrated device for measuring oxygen saturation proposed in this paper can support early detection of hypoxemia conditions. Further research is needed to certify the accuracy of the biosensor. This can be achieved through clinical research comparing an arterial blood gas tested in parallel to the measurement of the optical biosensor. However, from the novelty point of view, this work proves for the first time it is possible to extract SpO_2_ from a single wavelength.

## Figures and Tables

**Table 1:** Demographic and clinical data of the study group.

Subject	Sex	Age	Referenced SpO_2_ [%]	Biosensor’s SpO_2_ [%]
1	male	27	97	97.8
2	male	30	97	98.2
3	male	27	97	98.8
4	female	27	98	97.8
5	male	20	98	96.8
6	female	25	99	98.4
7	female	19	97	98.6
8	female	18	95	96.9
9	male	22	98	99.3
10	female	18	98	98.1
11	male	29	97	98.9
12	female	27	98	99.4
13	female	26	99	97.6
14	female	39	98	99.5
15	male	53	98	98.1
16	male	25	98	97.7
17	male	31	96	97.7
18	female	25	99	98.7
19	female	27	98	98.8
20	male	37	97	97.1
21	female	26	98	98.4
22	male	29	97	99.4
23	male	26	96	99.3
24	male	25	98	97.6
25	male	27	98	99.3
26	male	27	98	98.9
27	male	29	98	97.6
28	female	39	99	97.8
29	male	37	97	99.8
30	male	28	97	99.8
31	male	25	99	97.4
32	male	53	97	97.4
33	male	29	99	99.6
34	male	25	99	98.8
35	male	27	98	99.7
36	male	59	99	98.7
37	female	77	98	95.6
38	female	17	100	99.7

## Data Availability

The raw data supporting the conclusions of this article will be made available by the authors on request.
